# Review of the Electrical Characterization of Metallic Nanowires on DNA Templates

**DOI:** 10.3390/ijms19103019

**Published:** 2018-10-03

**Authors:** Türkan Bayrak, Nagesh S. Jagtap, Artur Erbe

**Affiliations:** 1 Institute of Ion Beam Physics and Materials Research, Helmholtz-Zentrum Dresden-Rossendorf, 01328 Dresden, Germany; t.bayrak@hzdr.de (T.B.); jagtap95@hzdr.de (N.S.J.); 2 Cluster of Excellence Center for Advancing Electronics Dresden (cfaed), TU Dresden, 01062 Dresden, Germany

**Keywords:** nanoelectronics, DNA origami, self-assembly, DNA metalization, DNA-origami metalization, electrical characterization

## Abstract

The use of self-assembly techniques may open new possibilities in scaling down electronic circuits to their ultimate limits. Deoxyribonucleic acid (DNA) nanotechnology has already demonstrated that it can provide valuable tools for the creation of nanostructures of arbitrary shape, therefore presenting an ideal platform for the development of nanoelectronic circuits. So far, however, the electronic properties of DNA nanostructures are mostly insulating, thus limiting the use of the nanostructures in electronic circuits. Therefore, methods have been investigated that use the DNA nanostructures as templates for the deposition of electrically conducting materials along the DNA strands. The most simple such structure is given by metallic nanowires formed by deposition of metals along the DNA nanostructures. Here, we review the fabrication and the characterization of the electronic properties of nanowires, which were created using these methods.

## 1. Introduction

The self-assembly and molecular recognition abilities of DNA may solve the problems of wiring and positioning at the nanoscale [1,2,3,4,5,6]. DNA templates can be used for a large number of applications ranging from sensing technology to nanocomputers due to the ease in fabrication of nanostructures of any complexity and the possibility for the deposition and controlled alignment of the structures on a substrate by molecular self-assembly. Single (ss) and double stranded (ds) DNA, DNA motifs and DNA origami techniques were developed that enabled the self-assembly of programmable DNA structures with complex forms as nanofabrication templates. The physical and chemical properties make DNA the perfect candidate for its use as interconnector down to dimensions on the order of 2 nm by building metallic nanowires and linking them to various nanomaterials such as semiconductor quantum dots, and metallic and magnetic nanoparticles. Using this scheme, manipulation of electrical and optical properties on the nanometer scale has been demonstrated in various fascinating experiments. For example, the fluorescence lifetimes of a quantum dot could be tuned by coupling it to metal nanoparticles in a well-defined geometry [7]. Moreover, voltage sensing was achieved by using voltage sensitive DNA origami structures, which convert voltages into optical signals [8]. These examples show that the precise positioning of nanoscale particles opens promising possibilities for the interconversion of optical and electronic signals at the nanoscale. This will make the development of true nano-optoelectronics possible. The ability of DNA self-assembly to form large structures has so far not been used for the fabrication of conducting networks. The main bottleneck in this context is given by the poor conductivity of single and double DNA strands [9]. Thus, the main challenge for the development of optoelectronic circuits is in the development of conducting structures formed via DNA nanotechnology. First steps towards this goal have been undertaken using metalized DNA nanostructures, which are the topic of the current review. Once this step has been successfully tackled, the further design of "integrated" circuits based on DNA will be possible.

Electrical characterization of nanowires has been performed by either contacting them directly with electrodes [10,11,12,13,14,15,16,17,18,19,20,21,22,23,24] or using scanning probe microscopy [25,26,27,28]. In most measurements, contact resistances dominated over the contribution from the resistance along the wire, and careful studies of the conductance of the wires indicated that the resistivity is higher than purely metallic resistivity. This may indicate that the growth mechanism, which leads to the formation of the nanowires, causes granularity of the metal leading to increased charge carrier scattering in the material.

In this review, we present DNA as templating material for metal nanoparticle building blocks, utilizing it for top-down metal deposition or direct metal chelating to form metallic nanowires. In the first part, we present a brief introduction of metal nanoparticle attachment and growth on DNA templates (Section 2). In the second part (Section 3), we review metallic nanowires and their electrical characterization. Finally, we will discuss in the conclusion which challenges and prospects of potential future applications can be identified. The resistance values and structural properties of DNA-templated nanowires, which have been fabricated by using different methods, have also been summarized in Table 1.

## 2. DNA Metalization

The DNA double helix consists of chemical nucleobases, Adenine (A), Guanine (G), Cytosine (C), and Thymine (T), deoxyribose sugars and phosphate groups. Nucleobases pair up as A and T or G and C to form units called base pairs bound through hydrogen bridges [29]. For the investigation of the electronic properties of DNA, typically ds-DNA, which is isolated from bacteriophage Lambda (λ-DNA) or from Calf Thymus is used. The first experiments on electronic transport through DNA nanostructures were performed under the assumption that the π-stacking of the conjugated base-pairs could conduct electricity. Wide ranges of conductivities were reported by theoretical and experimental studies [30]. Finally, DNA molecules with a length of l>40nm were observed to act as insulators, but below this length conductance could be observed that can be related to the base stacking of the ds-DNA. It has been demonstrated that stacks of CG-pairs act as relatively well conducting links along the DNA, while AT-pairs rather resemble tunneling barriers [31,32]. It is, however, challenging to generate stable conformations of DNA assemblies, which are exceeding the length of a few nanometers, with stacks of GC-pairs, only. Therefore, all DNA nanostructures, which have been generated so far, are electrically insulating. For the generation of nanostructures with electronic functionalities, conducting materials need to be added to the DNA nanomaterials. Especially for the creation of interconnects, the nanostructures need to be metalized for ohmic conductivity.

Several strategies have been followed for the metalization of DNA structures. The DNA structures can be activated by placing ions along the backbone of the DNA, which are then amplified using chemical methods. The activation can be also achieved by placing metal ions between the nitrogen atoms on nucleobases or π-stacked atoms. The DNA can be metalized directly by physical or chemical vapor deposition (PVD or CVD, respectively) methods as well. Metal nanoparticles can self-assemble into various structures by the interaction of functional groups, which are attached to their surface, with either the surface of other nanoparticles or a functionalized substrate. RNA or ss-DNA fragments can be used for the surface functionalization of the particles; the complementarity of these fragments with fragments on other nanoparticles or on, e.g., a DNA nanostructure placed on a surface, leads to the arrangement of the nanoparticles by self-organization. Typically, such self-organized metal structures need to be further treated in order to yield conducting nanostructures.

Here, we review in detail those methods that have been used for generating nanostructures for electronic purposes. Results of the subsequent electronic characterization will be discussed in Section 3 and are summarized in Table 1.

### 2.1. Activation by Metal Ions and Subsequent Metalization

**Figure 1:**
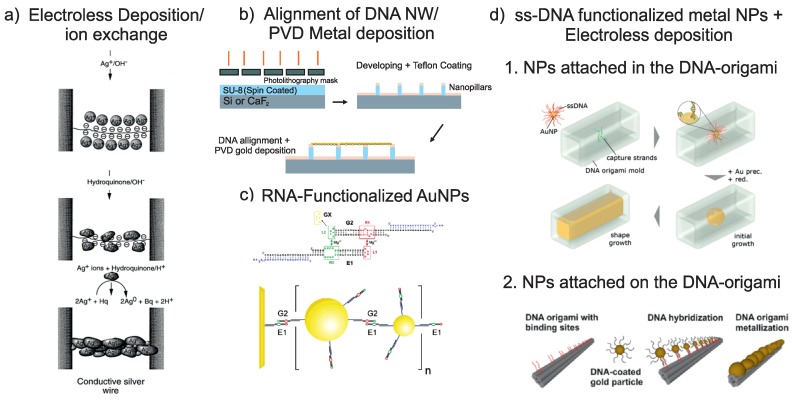
(**a**) conductive silver nanowire construction. Oligonucleotides attached to the gold electrodes and silver ions loaded to the oligonucleotides by Ag^+^/Na^+^ ion exchange. Wire developed using citrate solution and silver ions. Image taken with permission from [12]; (**b**) PVD deposition of metal on aligned DNA. The DNA is suspended on superhydrophobic pillars made from SU-8 resist by lithography; (**c**) RNA-functionalized nanoparticles (NPs) for the creation of conducting nanowires (NWs). The illustration shows the sequential assembly of AuNPs with diameters of 30 and 15 nm in diameter, and the controlled assembly of gold nanoparticle clusters connected to electrodes through RNA–RNA interactions. E1 and G2 are RNA hairpin molecules which interact through loop-receptor interactions L1–R1 and L2–R2. GX is a control RNA. Taken with permission from [41] (**d**) 1. DNA Origami molds filled with Au, with permission from [42]. 2. Nanoparticles NPs attached to six-helix bundles and subsequently enhanced. Taken with permission from [36].

**Figure 2:**
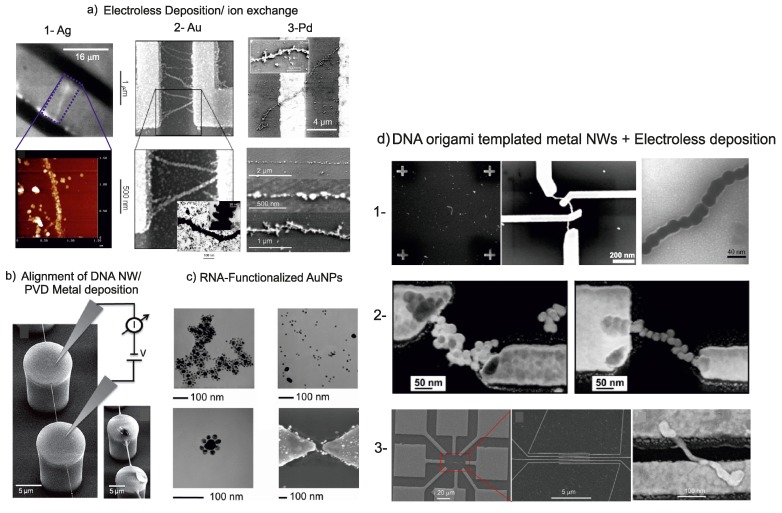
(**a**) images of nanowires made from Ag, taken with permission from [12], Au, taken with permission from [18], and Pd, taken with permission from [11]; (**b**) Scanning electron microscopy (SEM) image of suspended DNA nanowires metalized by evaporation of Au taken with permission from [20]. Thin (left) and thick (right) DNA nanowires NWs are bridging between two nanopillars. The scheme illustrating the electrical measurements setup which were performed by using micromanipulator on top of the nanopillars; (**c**) transmission electron microscopy (TEM/)SEM images of clusters of AuNPs bound together via RNA functionalization and contacted by electron beam lithography (EBL) (bottom right image) taken with permission from [41]; (**d**) various DNA templated Au nanowires contacted by EBL. (1) DNA molds filled with Au taken with permission from [38]; (2) DNA nanotubes metalized using functionalized AuNPs as seeds, images taken with permission from [36]; (3) Nanowires metalized by anisotropic growth of Au along Au nanorods, images taken with permission from [37].

Metal ions bound to DNA served as nucleation centers and catalysts for the growth of metal nanoparticles. Ions of various different metals, such as Ag [12], Pd [10], Au [33], Cu [26], and Rh [27], shown for example in Figure 1a and Figure 2a, have been coordinated on DNA nanostructures (e.g., ds-DNA, triple-crossover (TX)-DNA, DNA origami) to fabricate self-assembled nanowires. Understanding the importance of this activation step is crucial for achieving homogeneous metal layers during the subsequent growth. It has been shown by simulation and confirmed by experiments that increasing the activation time leads to an improved homogeneity of the metal layer. This points to the fact that the growth of metal on the DNA is nucleation limited [40].

The DNA mediated metal growth by using metal ions involves reduction and growth processes. In the reduction processes, reducing agents are added to the DNA solutions—examples are sodium borohydride (NaBH_4_) [25,27], hydroquinone, ascorbic acid [26], aldehyde [14,17], and dimethylamine borane (DMAB) [11,25]. Growth starting with those seeds or catalysts requires a subsequent reduction process for the enlargement of the metal clusters. In literature, chemical and electrochemical reduction processes were used in order to build wires which were characterized by two terminal *I*-*V*-measurements. A more detailed overview of the metal growth process on DNA templates is given in Section 2.4.

The metal seeding and growth can be performed both in solution and on a surface, where the DNA is already put in place for later electrical characterization. As an example, silver nanowires were fabricated by using microfluidic devices (made by PDMS) consisting of microchannels and gold electrodes. For this process, first, DNA molecules in solution were introduced into a microchannel. Then, DNA was electrostatically stretched between gold electrodes with an applied alternating electric field (1 MV/m at 1 MHz). For silver assembly on DNA, naphthalene diimide (NDI) molecules labeled with galactoses on both sides were introduced in the microchannel. The NDI intercalated in the ds-DNA, leaving the galactoses as reducing agents. At the last step, silver ions were injected and aldehyde group of the galactoses reduced the silver ions along DNA [23].

Metalization, which is formed inside the solution, is expected to yield more homogeneous layers than metalization of DNA on the substrate. A demonstration of this effect was given by doping the DNA with metal cations, which was done by mixing aqueous solutions of ds-DNA and metal salts [14]. Final DNA templated metallic structures were formed using reducing agents for the conversion of Metal (II) to Metal, for instance Cu(II) to Cu, also leading to subsequent growth of the metallic structures on the seeds. In the case of Cu-nanostructures, the resulting structures were then immobilized on a Si wafer and chemically or physically modified. Metal (II) concentrations resulted in the formation of dense networks due to association of the metal cations to the DNA causing effective charge neutralization of the DNA’s polyanionic phosphodiester backbone. The outcome of this charge neutralization was minimization of the repulsive electrostatic forces between DNA molecules. However, lower Metal(II) concentration was insufficient to promote aggregation forming more distinct 1-D structures. By controlling the reaction conditions, relatively smooth and continuous metal coating around DNA could be produced [26].

### 2.2. Placement of Metal Nanoparticles on DNA Nanostructures

Instead of using metal ions as catalysts for the start of metal growth, metal nanoparticles(NPs) have been bound to the DNA nanostructures as well. For example, pyridine modified gold nanoparticles have been assembled on the negatively charged backbone of ds-DNA templates. In order to form continuous and conductive gold nanowires on a substrate, the SiO_2_ substrate was treated by O_2_ plasma for ds-DNA immobilization on the surface, and a drop of pyridine modified gold NPs was deposited on the substrate. TEM images showed that the DNA templates were partially metalized by gold nanoparticles (AuNPs) following this protocol. As a final step, gold nanoparticles were enlarged by electroless deposition to form continuous wires [18].

Au rods and spherical AuNPs were used for selective deposition on DNA origami or TX-DNA tiles. Metal nanoparticles were functionalized by single stranded DNA with a specific sequence to selectively deposit in or on the DNA templates (Figure 1(d1,d2)). The experimental results were confirmed using a simple model for a binary mixture of DNA coated nanoparticles based on entropic cooperativity. Using this model, the dependence of metal coverage of the nanowires on factors like base-pair mismatch or ionic content of the solvent has been investigated [43]. Monte Carlo simulations have been performed for understanding and defining conditions for the crystallization of metallic nanoparticles [44].

The final construction yield of the DNA templated metallic NWs depend on three factors: DNA/DNA origami templates, metal nanoparticle attachment yield and metal growth. DNA, especially DNA origami templates, should be well formed, rigid (on the surface of Si/SiO_2_/Mica or any other surfaces) and stable after the particle attachment. High attachment yield of metal seeds on DNA/DNA origami is required for the formation of uniform, electrically conducting NWs. Typically, attachment yields for nanoparticles to DNA nanostructure are in the range of 10% to 50% [22,41] and have been confirmed using SEM (Scanning electron microscopy) or atomic force microscopy (AFM) studies. Clustering formation of the nanoparticles (steric hindrance), Coulomb interaction of nanoparticles (electrostatic repulsion), binding energy and individual nanoparticles bridging various binding sites (site bridging) are noted to be the main challenges for the particle assemble onto DNA templates [45]. To test the attachment probability, Takabayashi et al. [45] designed DNA origami nanotubes and nanorails with different numbers of binding sites, different numbers of capture strands per binding sites and different sequences of the capture strands. Experimental results have shown that the attachment probability does not depend on the sequences of the capture strands, which means that the binding energy is sequence independent. However, the attachment probability increased with the amount of capture strands per binding sites. High-density nanoparticle assembly on DNA origami templates can be achieved by carefully controlling the concentration of MgCI_2_ in buffer, the concentration of nanoparticles, and the hybridization time [35,46,47]. Metal nanoparticle growth on DNA templates is extensively discussed in Section 2.4. As discussed there and also in Section 3, the yield of metallically conducting wires is typically low, down to a few percent. The presented wires are merely to be seen as a proof of principle for the self-assembly of metallic nanostructures. In order to fabricate reliable structures, mainly the metalization process needs to be further improved.

However, DNA is recognized as a template for assembling different metal nanoparticles to continuous nanowires, precise positioning and immobilization of highly ordered DNA nanowires on solid substrates remain challenging. The formation of well-ordered homogeneous DNA nanowires was achieved by a flow-enabled self-assembly process [16]. With this process, arrays of high density long NWs could be fabricated over a length of 1.5 mm on a flat substrate. Exact positioning of NPs on top of DNA nanostructures has been achieved by using templates constructed via the DNA origami method [1]. DNA origami is a versatile and reliable method for synthesizing a large variety of shapes of organic nanostructures. This method is based on folding a long single stranded DNA scaffold into a desired shaped nanostructure through the use of synthetic staple strands as a complementary sequence. One of the advantages of the method is that nanoparticles can be site specifically attached by functionalizing the desired location of the origami structures either chemically or extending staple strands with additional nucleotides that serve as sticky ends for complementary sequences. DNA origami can be utilized as templates for the precise positioning of metal nanoparticles for fabrication of self-assembled metal nanowires, biosensors or plasmonic devices [47,48,49,50].

The first verification of increased conductance of a linear assembly of AuNPs on T-shaped DNA origami was achieved by using AuNPs with a diameter of 7.6 nm, which were functionalized with thiolated DNA sequences. The nanoparticles were self-assembled via extended staple strands on the T-shaped DNA origami in solution [34,37]. Au nanowires with a length of 120 nm and 240 nm were fabricated by using 11 and 22 AuNPs for 33 and 67 extended staple strands, respectively. The linear Au nanowires were deposited on a SiO_2_ substrate, which was treated by a plasma prior to deposition, in magnesium solution. TEM images showed that 12 nm gaps existed between assembled Au nanoparticles. Commercial Au plating solution with MgCI and HAuCI_4_ with NH_2_OH [51] were used to fill the gaps and fabricate continuous nanowires with incubation times varying from 1 min to 20 min. Substantial plating was done by 1 min commercial solution and 2 min HAuCI_4_ with NH_2_OH [52].

The beauty of the DNA origami method is that it is possible to design any shape of structures with nanoparticles of different metals in selected locations. After designing T-shaped structure and modifying them with AuNPs for fabricating linear nanowires, circular circuit (CC), nanotube, nanopillar and nanomold structures were fabricated by the DNA-origami method. By using CC-shaped DNA origami as a template and Pd seeds to modify the structure, continuous metal nanowires are formed by electroless deposition. This study is also important because it showed the first conductive Cu template DNA origami structures [35].

For the construction of metallic nanowires, 6-helix bundle DNA origami nanotubes were used as templates for AuNP assembly. Two bundles of six DNA origami and citride stabilized AuNPs (5 nm) were functionalized with ss-DNA sequences ((AAT)8T4-3 and 5 (ATT)3T4-3). An Au plating solution was used to enhance the size of the AuNPs after 10 min to 20 min incubation to homogeneous Au wires along the DNA origami nanotubes [36], as shown in Figure 1(d2).

Metalization of DNA origami structures has been achieved successfully. However, the generation of electrically conductive homogeneous metallic wires based on origami method is still challenging. In one study, Au rods with a length of 25 nm were used as seeds instead of spherical AuNPs to reduce the number of connection points along the linear DNA template (410 nm in length) and to improve the control of the width of the wires. TAE/Mg^+^^2^ buffer solution was used for the deposition of DNA origami on SiO_2_ and further Au rod deposition onto origami structures. The incubation time for seeding of the Au rods on the origami structures was in the range of 10 min to 1 h. Finally, the plating solution (a mixture of HAuCI_4_, CTAB, AgNO_3_, HCI, and ascorbic acid) was dropped on the surface further reducing the gaps to fabricate homogeneous gold nanowires. A reduction of the gap size was observed with long seeding times (1h) [37,39]. The origin behind the anisotropic gold growth process is given in Section 2.4.

### 2.3. Direct Metalization

Unspecific deposition of metal along DNA nanostructures can create metallic conductance of the resulting structures, as well. For example, an electron beam evaporation process has been used for the direct metalization of DNA templates. Since this method relies on the homogeneous deposition of metal on large area, it is mostly used for suspended DNA nanostructures [19]. DNA nanostructures, which are solidly attached to a substrate, would be electrically shorted when evaporated with a metal, unless very anisotropic deposition is granted and the height of the DNA nanostructure exceeds the height of the evaporated metal. Different substrates have been used for metal nanoparticle assembly on DNA structures and electrical measurements, such as Si, SiO_2_, glass and mica. Regular arrays of superhydrophobic nanopillars were used as a substrate for the alignment of λ-DNA. The nanopillars were fabricated using UV-lithography and a SU-8 resist [20]. Finally, instead of using metal nanoparticles for metalization of DNA by using self-assembly properties, a top-down metal evaporation method was used to coat the λ-DNA with a thin gold layer (30 nm thick). The resulting structures lead to high-quality, suspended nanowires, as shown in the sketch in Figure 1b and as a SEM image in Figure 2b.

### 2.4. Metal Growth on and in DNA Structures

After the initial placement of the metal seeds (ions or nanoparticles, see Section 2.1 and Section 2.2), metal needs to be further grown along the DNA nanostructure in order to form a continuous layer. A variety of methods exists for this step, which are based on, e.g., chemical reduction, photoreduction [53], electrochemical reduction [54] or direct deposition methods [20,55]. For the electrical characterization of the DNA nanostructures, mostly chemical reduction was used for the amplification of metal nanoparticles. Therefore, this technique will be discussed in more detail. Electroless plating is a catalytic process used for metal growth without the application of an external electric potential [56]. It is a redox reaction which is suitable for both conducting and insulating materials. To start the process, a catalytic surface or nucleation sites are required, as discussed above. This can typically be performed by activating the DNA nanostructure with ions [12] and subsequently growing the ions into nanoparticles, or by directly placing nanoparticles at well-defined positions defined by the functionalization of the DNA and the nanoparticles. The main step in electroless plating consists in the reduction of a metal ion at the position of a seed. The number and density of the seeds determines the size and the quality of the metal layers. The main disadvantage is given by the fact that the metal growth is not fully limited to growth on the DNA nanostructures. Seeds can form at other positions on the substrates, as well, leading to a large underground of metal in undesired locations. On top of this, the speed of the metal growth is difficult to control. Therefore, for an improved protocol of the growth, a better understanding of the process itself is mandatory.

During the growth of material on the DNA template, the surface tension of the metal and the line energy caused by the adhesion of the metal to the DNA template are competing. A model based on these energies has been used to derive that a low reaction stoichiometry leads to uniformly coated DNA templates rather than to the formation of isolated particles. In addition, an expansion of this model towards an implementation of the reaction kinetics has shown that for the formation of smooth nanowires sufficient annealing times have to be provided [57]. Such models may explain that experimentally observed nanowires formed by electrochemical amplification of nanoparticles tend to form chains of nanoparticles and thus yield comparably low conductance values [36].

An additional example for DNA mediated self assembled metallic nanowires are 2D triple-crossover (TX) DNA tiles modified with thiol containing oligonucleotides which were used to be transformed into DNA nanotubes as templates for Ag metalization. Metalization was performed in aqueous solution by mixing Ag seeds and DNA nanotubes in Mg^2+^ buffer, before the metal structures were deposited on a Si substrate [13].

Electroless plating mostly provides isotropic growth. It is, however, possible to achieve anisotropic growth of gold nanorod seeds immobilized on a DNA origami. In order to achieve this, a surfactant is added to the nanorod solution, which preferentially attaches to the 〈1 1 0〉 facets of the nanorods. The nanorods are oriented such that the 〈1 0 0〉 facets face each other, while the 〈1 1 0〉 facets are preferentially at the sides of the nanorods. Using this method, growth of the plating preferentially occurs in between the nanorods, while it is suppressed perpendicular to this direction [37,39].

## 3. Electrical Characterization of DNA-Based Metallic Nanowires

Once the DNA Origami has been positioned on a suitable, insulating surface and connected to metallic contacts or suspended between metallic electrodes, the electronic properties can be characterized. Several techniques have been developed for contacting the nanowires, thus combining bottom-up strategies with top-down contacting.

### 3.1. Lithographically Defined Contacts and In Situ/Ex Situ I-V Measurements

**Figure 3:**
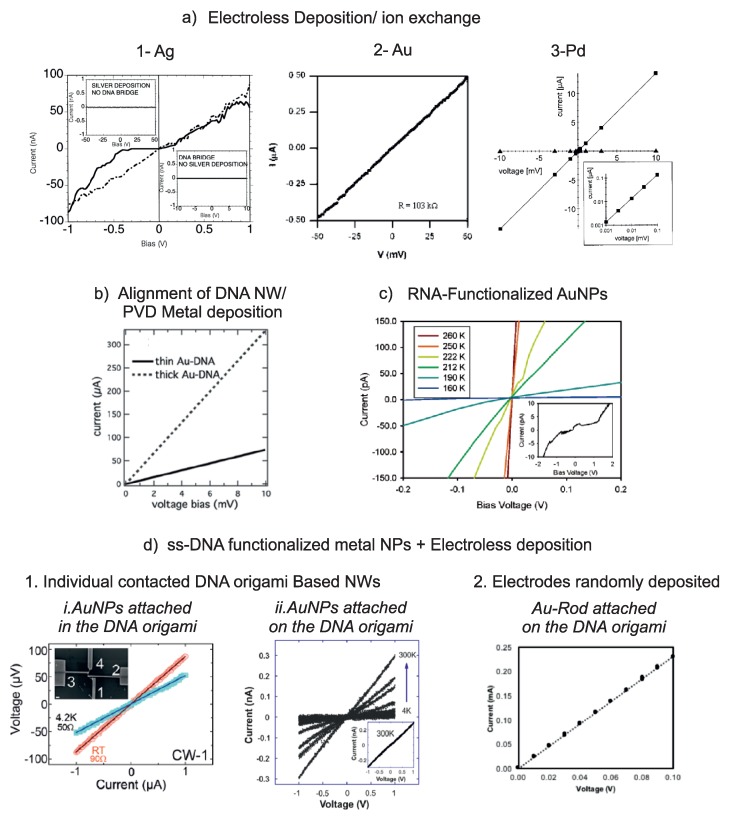
Examples of I(V)-curves taken for the nanowires shown in Figure 2. (**a**)wires that were created by activation and subsequent electroless deposition. Images are taken with permission from [12] (Ag), [18] (Au), and [11] (Pd); in case of Pd, a measurement after cutting the NW is shown (triangles). (**b**) electrical characterization of suspended nanowires which were metalized by direct Au evaporation. Image is taken with permission from [20]. Linear *I*-*V* curves at low bias prove the feasibility of Au coated DNA NWs for electrical circuits with resistances of around 30(140) Ω for thick (thin) NW; (**c**) electrical characterization of an array of AuNPs, which was formed by connections between RNA-functoinalized Au nanoparticles. I(V)-curves shows thermally activated charge transport. *I*-*V* curves at high temperatrues show linear behavior, however at 100 and 130 K characteristics can be compatible with single electron phenomena.Image taken with permission from [41]; (**d**) electrical characterization of nanowires formed on DNA Origami. Images are taken with permission from [38] (left), [36] (center), and [37]. Temperature dependent *I*-*V* characterization of origami mold templated AuNW (contacts 1 and 2 in the SEM image in the inset, 3 and 4 show insulating behavior) shows metallic conductance in *i*. Temperature dependent electrical characterization shows resistive wires of enhanced AuNPs on DNA nanotubes in *ii.*, which indicates the presence of small gaps between grown AuNPs. Anisotrophic nanorod growth in *2.* shows nanowires with resistances lower than 2 kΩ.

The first application of DNA double helices for the formation of metallic nanowires was done by metalizing DNA, which was bound to Au electrodes via sulfur-gold bonds, with silver [12]. The protocol was similar to the protocol described in Section 2.1. Two terminal *I*-*V*-measurements showed strongly nonlinear characteristics with a current suppression around zero bias and a hysteresis (see Figure 3a). While the gap around zero bias may be attributed to Coulomb blockade caused by the grainy structure of the wires, the hysteresis is more likely caused by electrochemical processes related to corrosion of the Ag nanoparticles. Differential resistance values were found in the range of 7 MΩ to 30 MΩ, and could be affected by the amount to which the Ag nanoparticles were grown.

Growth of various other metals was used for further metallic nanowires. Pd-coated DNA doublestrands were deposited on large Au electrodes defined by lithography. By chemical amplification of the Pd layers, continuous metal coatings of thicknesses in the range of 20 nm to 40 nm have been achieved [58]. The structure of the nanowires can be seen in the SEM images shown in Figure 2c. Wires, which were deposited without further treatment showed linear *I*-*V*-characteristics, see Figure 3(a3), with resistance values in the range of 5 kΩ, which were attributed to contact resistance. This contact resistance could be substantially reduced by depositing electron beam induced carbon lines on top of the nanowires on the Au pads. This leads to a pinning down of the nanowires to the contacts and an improved contact between wire and electrode. As a result, the overall resistance dropped to values below 1 kΩ [11,58].

Lithographically defined chromium/gold electrodes were patterned onto 5 μm long Ag wires created on TX-DNA tiles and three helix bundles for electrical characterization. Typical *I*-*V*-measurements showed ohmic behavior and resistance values in the range of 1 kΩ to 4 kΩ [13,17].

In order to create Au nanowires, negatively charged tris(hydroxymethyl)-phosphine-capped gold nanoparticles (THP-AuNPs) were bound densely to DNA and served as catalyst for th activation of the metal growth. Electroless gold plating of the resulting DNA-AuNP conjugates provided nanowires of size 30 nm to 40 nm in width and longer than ∼2μm with ohmic behavior and a resistivity of approx. 1 × 10^−5^ Ωm [28].

Ensembles of DNA doublestrands, which were decorated with pyridine-stabilized Au nanoparticles showed linear *I*-*V*-behavior at room temperature, see Figure 3(a2), and small bias voltages. The resistance of these wires was on the order of 100 kΩ and decreased with increasing times of the enhancement of the Au nanoparticles. In these measurements, the contribution of the contact resistance could not be quantified [18].

TX-DNA tiles decorated with Au nanoparticles were trapped between lithographically defined Au electrodes by dielectrophoresis. *I*-*V*-measurements were performed after the AuNP assembly and again after the amplification of the size of the AuNPs. As-deposited samples showed insulating behavior due to the large gaps between the AuNPs. After the chemical enhancement of the Au nanoparticles, a Coulomb blockade was observed in the *I*-*V*-curves with charging energies ranging from 0.25 eV to 2 eV. In particular, the large charging energies above 1 eV could not be related to the size of the nanoparticles. Therefore, a contribution of the grainy structure of the grown metal could not be excluded. Transport at low temperatures showed a decrease in noise due to the reduction of thermal fluctuations; the remaining switching noise, however, indicated the presence of charging noise in the underlying scaffold [22].

Pd-nanowires contacted with Au electrodes on SiO_2_ surfaces were measured at various temperatures and showed hints of the grainy structure of Pd generated via Pd reduction. The room temperature (RT) resistance of a single nanowire was on the order of 800 GΩ at 10 V. Measurements in a range from 150 K to 300 K showed an increase of the resistance with decreasing temperature, clearly deviating from purely metallic wires [10].

Ag-nanowires with a diameter of about 15 nm, which were generated using electroless chemical deposition on λ- and synthetic DNA, were contacted using lithographically defined Cr/Au electrodes. Measurements on these wires showed that initially high resistance values could be reduced by application of a bias voltage above 3 V to values below 1 kΩ. It can be assumed that an oxide or contamination layer, which has formed on the Ag nanowire, is destroyed by the applied voltage. Temperature dependent *I*-*V*-measurements of the voltage treated samples confirmed metallic conductance [14].

Contacts to suspended DNA nanostructures were formed by positioning them on insulating SU-8 pillars with metal contacts on top. These structures could be individually contacted using a microprober and electrically characterized. Metalization of the wires was done by using pvd methods. The charge transport mechanism of two individual wires (300 nm and 80 nm in diameter), which formed a bridge-like structure in between respective pillars, have been investigated. Ohmic behavior for low bias (at 10 mV) with 30 Ω and 140 Ω for thick and thin nanowires, respectively, was found, further increasing the voltage, the measured current saturated, and finally reaching the breakdown for the thin wire. SEM images of DNA bridges are shown in Figure 2b, and resulting *I*-*V*-measurements can be seen in Figure 3b [20].

Suspended nanowires, which were attached to lithographically defined gold electrodes by DNA combing during drying of the solvent, were characterized after deposition of gold layers. The resistance depends strongly on the resulting thickness of the wire after evaporation and was found to be, e.g., 7.7 Ω and 44.3 Ω for 80 nm and 60 nm width, respectively [19].

In order to detect genomic DNA from *E. coli*, ss-DNA could be built on an electrode and extended using rolling circle amplification (RCA). Using a variant of molecular combing, the resulting DNA strand was stretched between two electrodes with a separation of 5 μm and subsequently metalized by AuNPs, which were functionalized with complementary DNA fragments. The nanoparticles were amplified by either Ag(I) or Au(III) solutions and formed nanowires of 100 nm to 300 nm width. These wires were electrically characterized and showed a resistance in the kΩ (Ag) or Ω (Au) range [59]. In a different approach, a porous polycarbonate membrane, metalized on both sides was used to stretch long DNA oligonucleotides through the pores. These oligonucleotides were decorated with Au NPs, which were functionalized with complementary strands. Subsequent amplification of the metals lead to metallic nanowires, which were electrically characterized by contacting both sides of the membrane. The resistance depended on the amplification time of the nanoparticles and dropped to 10 Ω for times longer than 45 min [21].

In Figure 2c, SEM images of DNA functionalized AuNPs that have been interconnected via RNA bridges are shown. Temperature dependent measurement of the *I*-*V*-characteristics shown in Figure 3 indicated thermally activated transport by hopping [41].

### 3.2. Conductive AFM Measurements

Contacting single metalized DNA nanostructures has been reported using conductive Atomic Force Microscopy (c-AFM). In these measurements, a metalized AFM-tip is used to contact the metallic nanostructures at one end (or at both ends), and the current induced by an applied bias voltage is measured. The large advantage of this technique is that the contacted nanostructure can be imaged at the same time as it is measured electronically. This reveals additional information on the nature of the contact formed. In addition, the method is extremely flexible, since virtually any shape of nanowire can be contacted in this manner. Until today, DNA based Au, Pd, Cu, and Rh nanowires have been electrically investigated by c-AFM measurements [25,26,27,28].

The conductivity of Pd DNA nanowires was assessed by c-AFM. Two-terminal resistance was measured on single nanowires, which were sticking out of large nanowire assemblies on the sample surface. The tip of the c-AFM was then contacting the nanowire surface, and the second electrode was given by a fixed contact to a In/Ga eutectic contacting the assembly of the wires. Length dependent measurements indicated that contact resistance dominates the transport behavior of the wires. The resistance was in the range of 0.4 GΩ to 0.8 GΩ for DNA based Pd nanowires when prepared by the reduction of Pd(II) ions in dimethylaminoborane and 2 MΩ to 4 MΩ when NaBH_4_ was applied as a reducing agent [25].

Using similar methods, individual DNA/Cu structures were probed. A qualitative study using c-AFM indicated metallic conductivity of the Cu nanowires. Measurements using c-AFM showed an estimated resistance of about 100 MΩ. The determined resistivity value was two orders of magnitude higher than values obtained for Pd-seeded DNA-templated Cu nanostructures and six orders of magnitude higher than that of pure bulk Cu. Resistivity increases due to effects of grain boundaries and electron surface scattering as a result if thin Cu coatings were formed by this solution based synthetic approach [26]. X-ray diffraction studies with a Scherrer analysis offer two scenarios for the crystallite size in DNA templated metalic NWs [25,27,60]. For Pd, a crystallite size of 1.6 nm is found. This value is smaller than the nanowire heights observed by AFM (5 nm) [25]. However, the crystallite size of DNA templated Fe_3_O_4_ and Cu_2_O NWs show good agreement with AFM measurements [27,60].

Rhodium nanowires were fabricated using chemical and electrochemical reduction methods by using RhCl_3_·H_2_O as a source of Rh ions on a ds-DNA template. Conductive AFM measurements have been carried out on a SiO_2_ substrate. Resistance values as a function of relative distance have been shown for both Rh nanowires preparation. Resistivity values were found as 65 Ωcm and 41 Ωcm for chemical and electrochemical reduction method, respectively [27].

The fabrication of continuous Au nanowires, which are free of defects over several hundred nm, has been demonstrated by enhancing AuNPs, which were seeded on DNA templates. The enhancement procedure was performed under constant flow of HAuCl_4_ and ascorbic acid, which were mixed just before arriving at the sample surface. Both factors lead to an improvement of the homogeneity of the wires. Using this method, continuous wires with a diameter of 13 nm and a length >360 nm could be fabricated. The resistance of these wires was found to be on the order of 3 kΩ [33].

Electrochemical metal deposition has been used for the creation of superparamagnetic nanowires consisting of Fe. Using c-AFM, it could be shown qualitatively that the nanowires show electric conductivity. Magnetic force microscope (MFM) images indicate that the wires are superparamagnetic, indicating that they consist of separate Fe nanoparticles with a magnetization that directly follows an external magnetic field [54].

### 3.3. DNA Origami-Based Metal Nanostructures

In this section, a literature overview on the *I*-*V*-characterization of self-assembled metallic nanowires based on DNA origami templates is given.

Metalized DNA origami structures are typically fabricated on an insulating substrate, leading to a random distribution of nanowires on a surface. Contacts to the nanowires can be created by using finger like metallic electrodes deposited on a substrate without alignment [34,35,37]. Formation of contacts to DNA origami based wires can be see in in Figure 2(d3). In order to select individual nanowires and connect them to metallic electrodes, a method using a three step electron beam lithography process has been developed [36,38]

1. Gold alignment marks (with a mutual distance of ∼10μm in each direction) were fabricated on a SiO_2_ substrate.
2. SEM images were taken to register the location of the nanowires with respect to the alignment marks.
3. Electrical contacts to the individual wires were defined by EBL using the precise position measured in the SEM images.

*I*-*V*-measurements on T-shaped origami structures decorated with Au nanoparticles obtained resistances in the range of 1.5 kΩ to 2.3 kΩ [34].

Two terminal *I*-*V* measurements with −30 mV to 30 mV applied bias showed that the resistance of different numbers of CC-shaped Au metalized nanowires placed between electrode pairs was in the range from 1 kΩ to 5 kΩ. Cu wires showed relatively high resistances in the range of 40 kΩ to 1 MΩ for single bridge CC-shaped wires in gold electrode pairs. The Cu wires were not conductive again after two months [35].

Wires based on six-helix bundles were electrically measured by using two-terminal *I*-*V*-measurements from 4.2 K to 300 K. The resulting I(V)-curves are shown in Figure 3d. Resistance values were in the range between 100 MΩ to 2.8 GΩ at RT. The conductance was observed to decrease as a function of temperature. The temperature dependence could be attributed to conduction mechanisms such as hopping at high temperatures, thermionic emission at intermediate temperatures and tunneling at low temperatures. Activation energies at high temperatures have been calculated to be between 20 meV and 70 meV [36].

CC-shaped, T-shaped and nanotube structures modified with metal nanoparticles (Pd seeds or AuNPs with diameters of 5 nm to 8 nm) have been attached on top of origami structures. To achieve homogeneous metal nanowire formation, metal enhancers have been used. SEM and AFM images confirmed that nanoparticles/nanoseeds have been grown between 25 nm to 40 nm in diameter and the final length of the nanowires were in between 150 nm to 400 nm [35,36].

Instead of using metal seeds or spherical AuNPs on modified origami structures, the attachment of Au-rods followed by anisotropic electroless deposition has been used to improve metalization by clearly reducing the gap size between the grown AuNPs. *I*-*V*-characterization was performed on nanowires based on the deposition and growth of Au rods in between EBL defined Au electrodes at RT on SiO_2_ substrates. Resistance values of these wires were in the range of 0.435 kΩ to 20 kΩ. However, a small amount of the wires were very resistive (from 605 kΩ to 37 MΩ) [37,61]. I(V)-curves are given in Figure 3(d2).

Pillars of Au nanoparticles of diverse sizes were created by DNA directed programmable stacking into layer by layer DNA origami. For the investigation of potential charge transfer applications, a (3 + 3) pillar structure was deposited on SiO_2_ and the Au nanoparticles were enhanced by electroless deposition. Platinum electrodes from the nanopillars to gold contact pads were fabricated by using FIB deposition. *I*-*V*-measurements of nanopillars and bare SiO_2_ as control sample were performed in a voltage range from −10 V to 10 V. The *I*-*V*-curves were non-linear with *I* proportional to $V^{3}$ [24].

In a recent study, the possibility of creating DNA origami based continuous gold nanowires in a controllable array was shown. To this purpose, DNA mold monomers have been assembled into linear superstructures. Each monomer of the superstructure has two capture strands for AuNP assembly inside the hollow structure for subsequent metalization [42]. AuNP growth for obtaining a continuous conducting path along the superstructure has been done in solution. Two-terminal *I*-*V*-measurements have been performed at RT on 22 individually contacted nanowires with the contacting procedure given above. Temperature dependent charge transport measurements shown in Figure 3d revealed that a small part of the wires have shown metal-like conductivity [38].

## 4. Conclusions and Future Perspectives

Table 1 gives an overview on the metalized DNA structures, which have been reviewed in this paper. The wide spread in resistance values ranging from a few Ω up to several GΩ indicates that details in the formation of the metal layers determine the quality of the deposited metal layers. Both the length as well as the width of the wires seem to have only little influence on the resistance. Temperature-dependent measurements of the conductance seem to indicate that the main scattering mechanism leading to a metallic resistance which is much larger than observed for bulk metals is scattering at grain boundaries. More recent investigations indicate that this scattering can be avoided if the right growth conditions are developed. The reliability of these interconnects needs to be improved for future applications.

In the reported experiments, some key steps, which influence the electrical quality of the wires during growth, have been identified. For continuous metal coverage, the metal seeds need to be bound regularly to the DNA nanostructures. Thus, the activation time for ions and the deposition time for nanoparticles are important parameters for the metal growth. The growth during the amplification step can be limited by saturation effects, therefore repeated growth leads to better results than just a single step. During all deposition steps, the environment needs to be controlled in order to prevent aggregation of the materials and to enhance good adhesion to the surface for final deposition.

The main advantage of the use of DNA nanostructures as templates for electronic nanostructures is given by the ability to form complex structures on the nanoscale by self-assembly. Therefore, the initial study of metallic nanostructures, which are created using these methods, leads to important findings regarding feasibility and possible applicability of this self-organization scheme for the creation of nanoelectronics, even though the yield of working structures is currently low.

Once the creation of metallic interconnects has been accomplished, incorporation of active electronic building blocks will be investigated as the next step towards self-assembled nanoelectronics. This will open the way for the development of nanoelectronic circuits based purely on self-organization. The progress in self-assembly of metallic nanowires, which can be contacted with high precision and electronically characterized showing partly metallic conductivity, proves that the basic principle for this construction can certainly be achieved.

## Figures and Tables

**Table 1:** Summary of resistance values and construction properties of DNA templated metal nanowires.

DNA Building	Contact Method	NP	DNA Structure	Resistances	Metal Source/Metallization	Contact Metal	Substrate	Temp.	Height	Length	Width	References
DNA Metallization	EBL	Pd	λ-DNA	800 GΩ	Pd(Ac)_2_/Chemical Reduction	Cr/Au, Au, & Pd	Mica	120–300 K	NA	1–2 μm	7 nm	[10]
		Pd	λ-DNA	743 Ω and < 5 kΩ	Pd(CH_3_COO)_2_/Chemical Reduction	Au	SiO_2_	RT	NA	6.5 μm	50 nm	[11]
		Ag	λ-DNA/ds-DNA	30 MΩ, 7 MΩ	AgNO_3_/Chemical Reduction	Au	Glass	RT	NA	1.2 μm	100 nm	[12]
		Ag	TX-DNA	1.42–1.21 kΩ	AgNO_3_/Chemical Reduction	Cr/Au	Si	RT	(1.8±2) nm	16.5 nm	320 and 430 nm	[13]
		Ag	λ-DNA/ds-DNA	597 Ω–895 Ω (30 & 500 Ω at 77 K)	AgNO_3_/Chemical Reduction	Cr/Au	Si	77–300 K	NA	7 μm	15–35 nm	[14]
		Ag	DNA nanoribbons	200 Ω	Protein Array	NA	NA	RT	25 nm	5 μm	43 nm	[15]
		Ag	ds-DNA	500 Ω	AgNO_3_/Chemical Reduction	NA	PDMS transferred to Si	RT	NA	60 nm	NA	[16]
		Ag	TX-DNA	2.80 kΩ, 2.35 kΩ, 2.82 kΩ	AgNO_3_/Chemical Reduction	Cr/Au	Si	RT	35 nm	5 μm	40 nm	[17]
		Au	ds-DNA	103 kΩ	Pyridine modified gold nanoparticles/Gold-enhancer solution	Au	SiO_2_	RT	20 nm	1.25 μm	40 nm	[18]
		Au	λ-DNA/ds-DNA	44.3Ω (60 nm) and 7.7 Ω (80 nm)	E-beam Evaporation Gold	Ti/Au	Si/SiO_2_	RT	NA	800 nm	60 nm and 80 nm	[19]
		Au	λ-DNA	30–140 Ω	Thermal Evaporation Gold	Au	Pillars on Si or CF_4_ substrate	RT	5–350 nm in diameter	>5 mm	5–350 nm in diameter	[20]
		Au	ss-DNA	< 20 Ω	Gold nanoparticles/Gold-enhancer solution	Au	Polycarbonate memranes	RT	NA	10± 1.4 μm	NA	[21]
	AFM	Cu	ds-DNA	107 MΩ	Cu(NO_3_)_2_/Chemical Reduction	NA	TMS modified Si/SiO_2_	RT	11–20 nm	1.5 μm	20 nm	[26]
		Pd	λ-DNA/ds-DNA	0.4–0.8 GΩ with DMAB and 2–8 GΩ with NaBH_4_	K_2_PdCl_4_/Chemical Reduction	Au	SiO_2_	RT	NA	NA	5–45 nm diameter	[25]
		Au	DNA	2.4 kΩ	THP-AuNPs/Chemical reduction	Au	Si	RT	NA	2 μm	30–40 nm	[28]
		Au	DNA	3 kΩ to 1 GΩ	Au seeds/Chemical reduction	Au	Mica	RT	(10 ± 2, 13 ± 2 and 27 ± 3) nm	10–700 nm	25 nm	[33]
	AFM	Rh	λ-DNA	400–650 MΩ and 250–350 MΩ	RhCl_3_(H_2_O)/Chemical and electrochemical reduction	NA	SiO_2_	RT	3–31 nm in diameter	NA	3–31 nm in diameter	[27]
	Dielectro- phoresis	Au	TX-DNA tiles	Coulomb Blockade	DNA modified gold nanoparticles	Au	Si/SiO_2_	4.2–300 K	1.5 nm	50–60 nm	NA	[22]
	Micro- channel	Ag	ds-DNA	9 Ω	Chemical modification of gold nanoparticles	Au	PDMS	RT	NA	1 μm	40 nm	[23]
Metalized DNA Origami	EBL	Au	T-shaped	1.5–2.3 kΩ	DNA modified gold nanoparticles/Chemical Reduction	Au	SiO_2_	RT	NA	120 nm–240 nm	33 nm	[34]
		Pd	CC	1–5 kΩ for Au/40 kΩ–1 MΩ Cu	(NH_4_)_2_PdCl_4_/Chemical Reduction and Gold-enhancer solution	Au	Si	RT	NA	150 nm	35/30 for Au, 40 nm for Cu	[35]
		Au	Nanotube	116 MΩ–2.8 GΩ	DNA modified gold nanoparticles/Gold-enhancer solution	Ti/Au	SiO_2_	4.2K–300 K	40 nm	400 nm	30nm	[36]
		Au rod	Rectangular	435 Ω–36.9 MΩ	DNA modified gold rod/Chemical Reduction	Cr/Au	SiO_2_	RT	NA	< 410 nm	13–29 nm	[37]
		Au	Nanopillars	Highly resistive	DNA modified gold nanoparticles/Chemical Reduction	Pt	SiO_2_	RT	NA	NA	NA	[24]
		Au	Mold	90 Ω–30 GΩ	DNA modified gold nanoparticles/Chemical Reduction	Ti/Au	SiO_2_	4.2 K–300 K	20–30 nm in diameter	NA	20–30 nm in diameter	[38]
	EBID	Au rod	plus, cross, c-shaped	5.58kΩ–76 MΩ	DNA modified gold rod/Chemical Reduction	Cr/Au-Pd	Si	RT	NA	130 nm	12 nm	[39]

NP: nano particle; EBL: electron beam lithography; NA: not available; RT: room temperature; TX: triple-crossover; PDMS: Polydimethylsiloxane; AFM: atomic force microscopy; TMS: Tetramethylsilane; THP: Negatively charged tris(hydroxymethyl)- phosphine-capped; CC: circular circuit; EBID: electron beam induced deposition.
